# Effects of ultraviolet radiation and nutrient level on the physiological response and organic matter release of the scleractinian coral *Pocillopora damicornis* following thermal stress

**DOI:** 10.1371/journal.pone.0205261

**Published:** 2018-10-24

**Authors:** Lucile Courtial, Victor Planas Bielsa, Fanny Houlbrèque, Christine Ferrier-Pagès

**Affiliations:** 1 Sorbonne Universités, UPMC Université Paris 6, IFD-ED 129, France; 2 Centre Scientifique de Monaco, 8 Quai Antoine 1er, Monaco, Principality of Monaco; 3 UMR ENTROPIE (IRD, Université de La Réunion, CNRS), Laboratoire d’Excellence « CORAIL», BP A5, Nouméa cedex, New Caledonia, France; Helmholtz-Zentrum fur Ozeanforschung Kiel, GERMANY

## Abstract

Understanding which factors enhance or mitigate the impact of high temperatures on corals is crucial to predict the severity of coral bleaching worldwide. On the one hand, global warming is usually associated with high ultraviolet radiation levels (UVR), and surface water nutrient depletion due to stratification. On the other hand, eutrophication of coastal reefs increases levels of inorganic nutrients and decreases UVR, so that the effect of different combinations of these stressors on corals is unknown. In this study, we assessed the individual and crossed effects of high temperature, UVR and nutrient level on the key performance variables of the reef building coral *Pocillopora damicornis*. We found that seawater warming was the major stressor, which induced bleaching and impaired coral photosynthesis and calcification in all nutrient and UVR conditions. The strength of this effect however, was nutrient dependent. Corals maintained in nutrient-depleted conditions experienced the highest decrease in net photosynthesis under thermal stress, while nutrient enrichment (3 μM NO_3_^-^ and 1 μM PO_4_^+^) slightly limited the negative impact of temperature through enhanced protein content, photosynthesis and respiration rates. UVR exposure had only an effect on total nitrogen release rates, which significantly decreased under normal growth conditions and tended to decrease also under thermal stress. This result suggests that increased level of UVR will lead to significant changes in the nutrient biogeochemistry of surface reef waters. Overall, our results show that environmental factors have different and interactive effects on each of the coral’s physiological parameters, requiring multifactorial approaches to predict the future of coral reefs.

## Introduction

Anthropogenic climate change has emerged as a serious global-scale threat to the structure, functions and viability of coral reef ecosystems [1, 2, 3]. In particular, reefs in shallow waters are facing increases in seawater temperature, ultraviolet radiation (UVR) levels and storm events frequency [4, 5]. Beyond their direct effects on reefs, such environmental changes are responsible for variations in nutrient concentrations. For instance, heat waves enhance nutrient depletion through water column stratification [6], while storms can induce water-column mixing and nutrient upwelling [7]. On top of these global changes, coastal reefs suffer from local pressure, such as overfishing, sedimentation and eutrophication (increased supply in organic and inorganic nutrients) [8]. Although healthy, thriving coral reefs do occur in a broad range of environmental conditions and nutrient availabilities [9, 10], alterations of the surface water conditions have often led to the decline of scleractinian corals, the major reef builders [2, 9, 11]. Over the last decade, we observed an increase in the frequency and the intensity of coral bleaching event (*i*.*e*. massive loss of symbionts or photosynthetic pigments from the host tissue), responsible for high coral mortality [11, 12].

Coral bleaching, the most pressing issue for tropical coral reefs on a global scale [12], is often associated with high temperature anomaly events. Local conditions may also influence coral resilience to elevated temperatures by exacerbating or mitigating its detrimental effects [13, 14]. Because of the complexity of coral response to each factor and the multiple interactions that exist between them (additive, synergistic or even antagonistic interactions [15], the combined effects of these factors on coral physiology are not yet well understood. In particular, several UVR and nutrient levels can occur in a reef during a thermal stress event. Nutrient level can vary with the eutrophication status of the reef, but also with rainfalls, floods, or local biological activity depleting nutrients from surface waters [16, 17], while UVR level reaching a coral colony depends on the local shading, the surface orientation, the cloud cover or the depth at which corals grow [18, 19]. Results from previous studies showed different responses to the combination of thermal stress with UVR [20, 21, 22, 23, 24, 25] or with nutrient enrichment [14, 26, 27], depending on the environmental context, as well as the quality and severity of the stress. For example, different species of nitrogen (e.g., nitrate, ammonium, urea), may have very different effects on individual compartments of the coral holobiont, which may ultimately affect overall holobiont functioning [26, 28]. All together, the results obtained call for further studies on the individual and interactive effects of these factors to better forecast the ecological consequences of global warming on reef corals.

Corals are also known to release large amounts of organic matter (OM) in particulate and dissolved forms into the surrounding seawater [29], and release rates can be largely impacted by environmental changes. As OM is a main nutritional source for reef microorganisms [30], modifications in OM quality and/or quantity can affect reef water biochemistry, microbial community composition and activity and may ultimately even trigger virulence in reef-associated microbes [31, 32]. Despite this key role of OM, very little information is available on species-specific OM release rates under thermal stress [33, 34], UVR exposure and nutrient availability [35].

In this study, we describe the individual and combined effects of thermal stress, UVR exposure and nutrient (nitrate, phosphate) enrichment/depletion on the key performance variables of the reef-building coral *Pocillopora damicornis*. The first aim was to test whether the combination of multiple stressors (i.e. UVR and nutrient levels) reduces coral resistance to temperature- induced bleaching. The second aim was to investigate the effect of multiple environmental parameters (temperature, UVR and nutrients) on the release rates of OM. Interactions of multiple stressors, and the resulting cumulative impacts, have been identified as a research priority by management bodies and researchers (summarized in [15]). The results obtained will help assessing whether local conservation efforts (such as reduction in urban runoff) could significantly enhance the ability of corals to withstand the effects of climate change and could avoid the emergence of coral diseases.

## Material and methods

Ten mother colonies of *Pocillopora damicornis* (Pocilloporidae), originating from the Pacific (CITES: FR1602900021-E), were grown at 25°C in the aquaria set up of the Centre Scientifique de Monaco and fed twice a week with *Artemia salina* nauplii. They were maintained for several months in flow through aquaria, continuously supplied with oligotrophic seawater (~0.5 μM NO_3_^-^ and ~0.1 μM PO_4_^+^), with a water renewal of 20L h^-1^. They were kept under a PAR (photosynthetically active radiation) of 200 μmol photons m^-2^ s^-1^ provided by 400 W metal halide lamps (HPIT, Philips). The radiation intensity was controlled using a LI-COR data logger (LI-1000) connected to a spherical quantum sensor (LI-193) for PAR and using an International Light ILT1400 portable radiometer and detectors (SEL240/UVB-1/TD and SEL033/UVA/TD) for UVR. A total of 60 nubbins (ca. 3 cm diameter) were collected from 10 mother colonies (6 nubbins per colony). After being cut, nubbins were allowed to heal under the same conditions as the mother colonies for four weeks and then kept unfed for three weeks before the start of the experiment.

### Experimental settings

After the seven weeks of preparation, nubbins were evenly distributed in twenty-four tanks of 25 L, continuously supplied with seawater. Twelve conditions, representing two UVR levels, two temperatures, and three nutrient conditions in a factorial design were tested in duplicated tanks: nutrient-depleted seawater (<0.1 μM nitrate (NO_3_^-^) and <0.05 μM phosphate (PO_4_^+^)) without UVR (hereafter called D0UV) and with UVR (DUV); ambient seawater (~0.5 μM NO_3_^-^ and ~0.1 μM PO_4_^+^) without UVR (i.e. control condition, C0UV) and with UVR (CUV); enriched seawater (3 μM NO_3_^-^ and 0.5 μM of phosphates) without UVR (E0UV) and with UVR (EUV). The six conditions were maintained at ambient temperature (AT, 25°C ± 0.3) or high temperature (HT, 31°C ± 0.3). PAR remained at 200 ± 10 μmol photons m^-2^ s^-1^ inside the tanks (12: 12 light: dark). UVR was provided 4 h a day by Q-Panel UVA 340 lamps and was adjusted to match the mean levels measured at 1–5 m depth on reefs [36], ca. 1.5 W m^-2^ UVB and 30 W m^-2^ UVA. As corals were grown without UVR prior the experiment, their response can be compared to deep-living or shaded corals facing an increase in UVR level. Temperature was controlled using heaters connected to ElliWell PC 902/T controllers. Seawater in the tanks was renewed at a flow rate of 20 L h^-1^. Nutrient depleted tanks were continuously supplied with water from batch tanks depleted in nutrients with a bio-chemical filter (Fluidize bed filter BF-700). The filters were installed following the manufacturer’s recommendation several weeks prior the experiment. In nutrient enriched conditions, tanks were supplied with a solution of sodium nitrate (NaNO_3_) in combination with sodium dihydrogenophosphate (NaH_2_PO_4_) continuously pumped from batch tanks with a peristaltic pump and delivered to the experimental tanks at a final concentration of 3 μM for NaNO_3_ and 0.5 μM for NaH_2_PO_4_. The stock solutions were renewed every 3 d and added to the tanks at a constant flow rate of 0.3 L h^-1^. Nutrient concentrations in each aquarium were monitored in triplicate once a week using an Autoanalyzer (Alliance Instrument, AMS, France) according to [37].

The experiment was run for a total of 9 weeks. During the first 4 weeks, all nubbins were acclimated to the UVR and nutrient conditions. The temperature was then gradually increased (within a week) from 25°C to 31°C in HT tanks. High temperature was maintained for 3 additional weeks before the measurements were conducted. At the end of the 9 weeks, 5 nubbins per condition, randomly taken in the replicated tanks, were used to measure photosynthesis, respiration and calcification rates, total organic carbon (TOC) and total nitrogen (TN) fluxes and extracellular α-glucosidase and aminopeptidase activities. At the end of the measurements, nubbins were frozen at -20°C to assess the symbiont density, chlorophyll *a* (chl *a*) and protein contents. For all measurements, temperature, UVR, light and nutrient conditions were maintained constant and identical to the experimental tanks except for the short-term measurements of photosynthesis, which were made without UVR. Coral surface area, measured using the wax coating technique [38], were used to normalize the data.

### Physiological measurements

Nubbins were incubated in 50 mL glass chambers filled with water from the different nutrient conditions, filtered at 0.45 μm and continuously stirred with stirring bars. Each chamber was equipped with oxygen-sensors (Unisense optode) connected to the Oxy-4 software (Chanel fiber-optic oxygen meter, Presens, Regensburg, Germany). Two calibrations of the optodes (100% and 0% oxygen) were done prior each measurement using air-saturated and nitrogen saturated seawater. Rates of net photosynthesis (Pn) (i.e. true photosynthesis minus photorespiration and dark respiration, [39]) was first assessed at 200 μmol photons m^-2^ for 30 minutes. Light was stopped and rates of respiration were then measured in the dark, to approach as much as possible the photorespiration [40, 41, 42]. Pn and R were deduced from regressing oxygen production or consumption against time. Gross photosynthesis (Pg) was calculated by adding the absolute values of R to Pn. Data were expressed in μmol O_2_ h^-1^ cm^-2^ and corrected against a blank (filtrated seawater incubated for the same period without nubbin). Calcification rates were assessed using the buoyant weight technique [43] on 5 nubbins per treatment the week before the beginning of the experiment and after the 9 weeks of treatments. As tissue of *P*. *damicornis* accounted for only 1.2% of the total weight (checked according to [44]), buoyant weight indeed assessed the skeletal growth (calcification) of this coral species. Calcification in % d^-1^ was calculated using the equation: (*BW_f_* − *BW_i_*)/(*BW_i_* × *Time*) × 100 where *BW_f_* and *BW_i_* are the final and the initial buoyant weight respectively and Time is the number of days between the two measurements.

### Symbiont, chlorophyll a and protein content

Coral tissue was extracted from the skeleton using an air pick and homogenized with a Potter tissue grinder. The symbiont density was then quantified with three sub-samples of 100 μL using a Z1 Coulter Particle Counter (Beckman Coulter) [45]. Proteins were extracted according to [46] in sodium hydroxide and quantified using a BCA essay kit [47] on a Xenius spectrofluorometer (Safas, Monaco). To quantify chl *a*, the remaining homogenate was centrifuged at 5000 x *g* for 10 min at 4°C to separate the host tissue (supernatant) from the symbionts (pellet) and the pellet was re-suspended in 5 mL of acetone. Samples were then kept at 4°C for 24 h in darkness to insure total extraction. The next day, samples were centrifuged for 15 min at 11000 x *g* and the absorbance was measured at 630, 663 and 750 nm using a Xenius spectrofluorometer. Chl *a* concentrations were calculated using the equations of [48]. All data were normalized to the skeletal surface area, (cm^2^) of each nubbin, using the wax-dipping method [49].

### Organic matter release

To assess the total amount of organic carbon (TOC) and nitrogen (TN) released by corals as well as the extracellular enzymatic activity (EEA), nubbins were incubated for 6 h in 250 mL of 0.45 μm-filtered seawater, taken from the different nutrient conditions. Blanks (seawater without nubbin) were run in parallel. For measurements of TOC and TN concentrations, triplicates of 20 mL of seawater from each beaker were sampled with sterile syringes at the beginning and the end of the incubation. Samples were stored at -20°C in glass vials, previously washed for 24 h in 10% HCl and burned at 500°C for 4 h [29], before being analyzed using a TOC-L analyzer (Shimadzu, Japan). Fluxes (TOC or TN, nM h^-1^ cm^-2^) were calculated as:

$$TM=\frac{{TM}_{coral}-{TM}_{blk}}{Surface\times Time}$$

where TM designates TOC or TN, TM_coral_ and TM_blk_ (nM h^-1^) are the TM concentrations in the seawater after 6 h incubation respectively with or without nubbin, Time (h) is the 6 h incubation time and Surface is the nubbin surface (cm^-2^). Positive fluxes indicate net release of OM, while negative fluxes indicate net uptake of organic matter by corals.

### Statistical analysis

All data were expressed as mean ± standard deviation and calculations were performed using R statistical framework (R version 3.4.3). All independent variables were coded as numerical variables according to Table 1.

**Table 1:** Code corresponding to the numerical data and used in the generalized linear model approach.

Low Temperature	High Temperature	Nutrient depletion	Control	Nutrient enrichment	Low UVR	High UVR
0	1	0	1	2	0	1

Nutrient-depleted and nutrient-enriched conditions were analysed separately, because very different mechanisms may be involved in the response of corals to each nutrient condition, and we cannot assume a linear relation (or even a monotonous relation) from nutrient depleted to control and nutrient-enriched conditions. Therefore, a first analysis considered the individual and combined effects of temperature (T), UVR (UV) and nutrient (Nut)-depletion on the main physiological traits of *P*. *damicornis* (we ignored the comparison between nutrient-enriched and control conditions). A second analysis was performed to assess the effects of temperature, UVR and nutrient-enrichment on *P*. *damicornis* (we ignored the nutrient-depleted vs control comparison).

For each nutrient condition, data were analysed using a Generalized Linear Model (GLM) approach [50] with multiplicative effects, according to the following equation:

$$E[y]=\beta_{0}+\beta_{1}(T)+\beta_{2}(Nut)+\beta_{3}(UV)+\beta_{4}(T\times Nut)+\beta_{5}(T\times UV)+\beta_{6}(Nut\times UV)+\beta_{7}(T\times Nut\times UV)$$

where y is the measured response (physiological and organic matter fluxes), and βi are the maximum likelihood coefficients. A model selection procedure was used based on Akaike Information Criteria corrected for small samples (AICc). For each response variable, seven models were computed (T, Nut, UV, T*Nut, T+UV, Nut*UV, T*Nut*UV) and AICc, Delta AICc and the Akaike weights were calculated. In addition, when the analyses showed a significant positive interaction between two-or three environmental factors, we characterized this combined effect as “synergistic”, because it exceeds the individual effects. On the contrary, if the combined effect of the stressors is equal or less to the sum of the individual effects, the interaction has been considered as additive and antagonistic respectively [15].

#### Outliers treatment

Once a model has been selected, Cook’s distance [51] was used to identify possible outliers in each of our measured responses. We considered outliers, all points for which Cook’s distance is above 4 standard deviations from the mean. After removal of outliers, a new model selection procedure was performed to confirm the best model. For each nutrient condition, a correlation matrix and a Principal Component Analysis (PCA) were also calculated, after removing outliers. Since each factor has different non-comparable units, we normalized the data prior to building the PCA.

## Results

### Global analysis on coral physiology and organic matter fluxes

**Fig 1:**
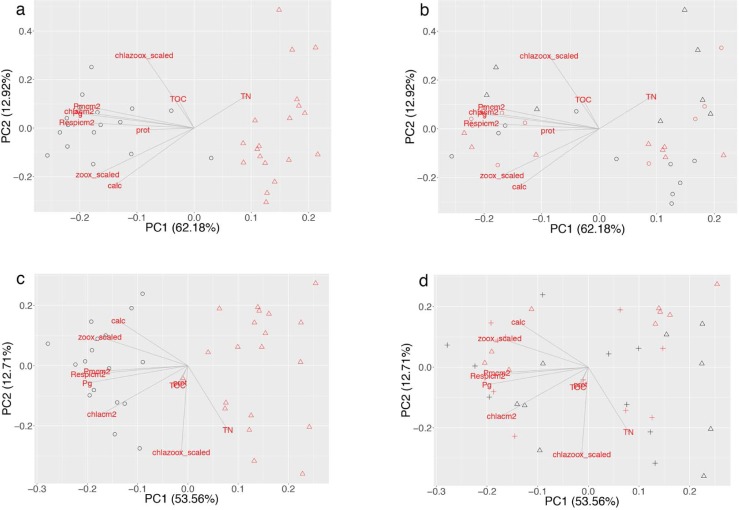
Distance biplots resulting from the Principal component analyses. Physiological traits (black) used as descriptors represent symbiont density (Symbiont), protein (Protein) and chlorophyll a (Chla) contents, calcification rates (Growth), respiration (R) and net photosynthetic rates (Pn), total organic carbon (TOC) and total organic nitrogen (TN) fluxes. **Fig 1a, b: nutrient depleted versus control conditions.** The first PCA axis accounts for 62.2% of the variability and the second PCA axis for 13.0%. Fig 1A: black and red symbols represent nubbins at 25°C and 31°C respectively; Fig 1B: circles and triangles represent nubbins maintained in depleted and control conditions, while black and red symbols represent no UVR and UVR condition, respectively. **Fig 1c, d: nutrient enriched versus control conditions.** The first PCA axis accounts for 62.2% of the variability and the second PCA axis for 13.0%. Fig 1C: black circles and red triangle symbols represent nubbins at 25°C and 31°C respectively. Fig 1D circles and triangles represent nubbins in nutrient-depleted and control conditions, while black and red color represent no UVR and UVR condition respectively.

For the nutrient-depleted and nutrient-enriched analyses, only the first component of the PCAs is significant, and explains ca. 62% and 54% of the variance respectively (Fig 1A and 1C). This component shows a temperature effect on corals, and separates nubbins growing at 25°C (black symbols) and 31°C (red symbols). Overall, no clear effect of UVR or nutrient level is detected by the PCA (Fig 1B and 1D), suggesting that temperature was the main factor controlling coral physiology.

### Physiological changes

Results of the Generalized Linear Model approach (Tables 2–4) show that the observed changes in the key physiological parameters of *P*. *damicornis* were mostly explained by temperature and nutrient levels, alone or in combination. On the contrary, UVR exposure had fewer individual or combined effects. Therefore, for clarity, we will first discuss and present the overall effects of temperature and nutrient levels regardless of UVR exposure, and then focus on the few interactions with UVR.

**Table 2:** Results of the generalized linear model approach on the effects of temperature and UVR on corals maintained in the control condition.

Variable	Temperature	UV	Temperature*UV
calcification	-1.9249 (p = 0.0026**)	0.251 (p = 0.6493)	0.9507 (p = 0.2323)
symbiont density	-0.8121 (p = 0.0027**)	0.1785 (p = 0.4479)	0.2263 (p = 0.4955)
areal chlorophyll content	-0.7069 (p = 0.0123*)	0.4022 (p = 0.1279)	-0.3563 (p = 0.3295)
chlorophyll/symbiont	0.1056 (p = 0.7648)	0.2237 (p = 0.5284)	-0.582 (p = 0.2531)
protein	0.2687 (p = 0.3997)	0.4395 (p = 0.1761)	-0.3861 (p = 0.3922)
areal respiration rates	-2.4591 (p = 2.12e-08 ***)	0.2195 (p = 0.3768)	-0.2895 (p = 0.409)
areal gross photosynthesis	-2.6146 (p = 6.75e-09 ***)	0.1698 (p = 0.4834)	-0.7424 (p = 0.0414)
areal net photosynthesis	-2.0096 (p = 2.52e-05 ***)	0.0558 (p = 0.8732)	-0.1994 (p = 0.6875)
total organic carbon release rate	-0.606 (p = 0.2921)	-0.4856 (p = 0.3956)	-0.1002 (p = 0.9002)
total nitrogen release rate	0.0282 (p = 0.6821)	**-0.133 (p = 0.0667)**	0.0102 (p = 0.9163)

**Table 3:** Results of the generalized linear model approach for the nutrient-depleted corals compared to control conditions. All data are normalized to the surface area of the corals.

Nutrient depleted vs Control				
Variable	Best model	Temperature	Nutrient level	UV
calcification	Temperature*Nutrient	-0.34 (0.38) p = 0.38	0.17 (0.38) p = 0.65	-
symbiont density	Temperature*Nutrient*UV	-0.725 (0.1431) p = 1.77e-05***	-0.418 (0.1431) p = 0.00645 **	-0.158 (0.1431) p = 0.27914
areal chlorophyll content	Temperature*Nutrient	-1.377 (0.1311) p = 2.33e-12 ***	-0.062 (0.1347) p = 0.6497	-
chlorophyll/symbiont	Temperature*Nutrient*UV	**-0.519 (0.2339) p = 0.0339** *	**0.518 (0.2480) p = 0.0450** *	**0.496 (0.2339) p = 0.0419** *
protein	Temperature	**-0.246 (0.1136) p = 0.0369** *	-	-
areal respiration rates	Temperature+Nutrient	-2.656 (0.0790) p = <2e-16 ***	0.146 (0.0790) p = 0.0723.	-
areal gross photosynthesis	Temperature*UV	-2.593 (0.152) p = < 2e-16 ***	-	0.235 (0.1522) p = 0.131
areal net photosynthesis	Temperature*Nutrient	-1.375 (0.2183) p = 2.82e-07 ***	0.722 (0.2184) p = 0.00216 **	-
total organic carbon release rate	Temperature	-0.162 (0.207) p = 0.438	-	-
total nitrogen release rate	Temperature + UV	0.075 (0.0211) p = 0.00111 **	-	**-0.053 (0.0211) p = 0.01650** *
**Variable**	**Temperature:Nutrient**	**Temperature:UV**	**Nutrient:UV**	**Temperature:Nutrient:UV**
calcification	**-1.114 (0.5356) p = 0.0447** *	-	-	-
symbiont density	-0.087 (0.2024) p = 0.6710	**-0.464 (0.2024) p = 0.02881** *	0.611 (0.2087) p = 0.00635 **	0.4158 (0.2907) p = 0.16261
areal chlorophyll content	0.353 (0.1879) p = 0.0685.	-	-	-
chlorophyll/symbiont	0.292 (0.340) p = 0.3976	0.199 (0.330) p = 0.5523	-0.605 (0.340) p = 0.0858.	-0.44840 (0.47503) p = 0.3525
protein	-	-	-	-
areal respiration rates	-	-	-	-
areal gross photosynthesis	-	**-0.55 (0.215) p = 0.015** *	-	-
areal net photosynthesis	**-0.735 (0.3088) p = 0.02277** *	-	-	-
total organic carbon release rate	-	-	-	-
total nitrogen release rate	-	-	-	-

* represents an interaction while + represents a single effect.

**Table 4:** Results of the generalized linear model approach for the nutrient-enriched corals compared to control conditions. All data are normalized to the surface area of the corals.

Nutrient-enriched vs Control				
Variable	Best model	Temperature	Nutrient level	UV
calcification	Temperature	-1.011 (0.284) p = 0.00102 **	-	-
symbiont density	Temperature*Nutrient*UV	**-0.637 (0.321) p = 0.056196.**	0.580 (0.1435) p = 0.000327 ***	1.2665 (0.3366) p = 0.001 ***
areal chlorophyll content	Temperature*Nutrient	-1.64527 (0.35171) p = 4e-05 ***	-0.0265 (0.1581) p = 0.87206	-
chlorophyll/symbiont	Temperature*Nutrient	**-1.001 (0.4177) p = 0.02208** *	**-0.3577 (0.1878) p = 0.06506.**	-
protein	Nutrient	-	0.3582 (0.1029) p = 0.001296 **	-
areal respiration rates	Temperature*Nutrient	-3.2868 (0.25281) p = 6e-15 ***	-0.0507 (0.1137) p = 0.6583	-
areal gross photosynthesis	Temperature*Nutrient*UV	-3.3330 (0.3715) p = 4e-10 ***	0.2488 (0.1680) p = 0.1487	-0.1197 (0.3715) p = 0.7495
areal net photosynthesis	Temperature*Nutrient	-0.1755 (0.5698) p = 0.760	2.172 (0.2548) p = 3.7e-10 ***	-
total organic carbon release rate	Temperature	-0.3570 (0.2721) p = 0.198	-	-
total nitrogen release rate	UV	-	-	-0.0966 (0.0345) p = 0.008**
**Variable**	**Temperature:Nutrient**	**Temperature:UV**	**Nutrient:UV**	**Temperature:Nutrient:UV**
calcification	-	-	-	-
symbiont density	-0.1754 (0.2030) p = 0.394302	-0.6490 (0.4651) p = 0.172824	-0.8134 (0.2092) p = 0.000499 ***	0.6007 (0.2915) p = 0.047*
areal chlorophyll content	0.6218 (0.2207) p = 0.00789 **	-	-	-
chlorophyll/symbiont	0.6552 (0.2621) p = 0.01725*	-	-	-
protein	-	-	-	-
areal respiration rates	0.5541 (0.1586) p = 0.00131 **	-	-	-
areal gross photosynthesis	**0.4732 (0.2309) p = 0.0489** *	-0.8264 (0.5132) p = 0.1175	0.0443 (0.2309) p = 0.8491	0.3291 (0.3217) p = 0.3142
areal net photosynthesis	-1.934 (0.3604) p = 4.89e-06 ***	-	-	-
total organic carbon release rate	-	-	-	-
total nitrogen release rate	-	-	-	-

* represents an interaction while + represents a single effect.

#### Effects of temperature and nutrient level

**Fig 2:**
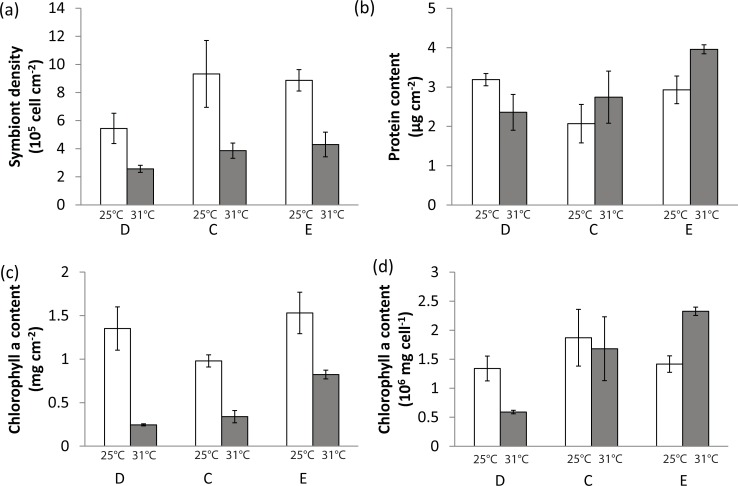
Effect of temperature and nutrient level in absence of UVR exposure. Symbiont density (cell cm^-2^, a), protein content (mg cm^-2^, b), chlorophyll a content per surface area (mg cm^-2^, c) and chlorophyll a content per symbiont cell (10^6^ mg cell^-1^, d) in nutrient depleted (D), control (C) and nutrient enriched (E) condition at 25°C (white columns) and 31°C (grey columns). Error bars represent standard deviations calculated from five replicates.

**Fig 3:**
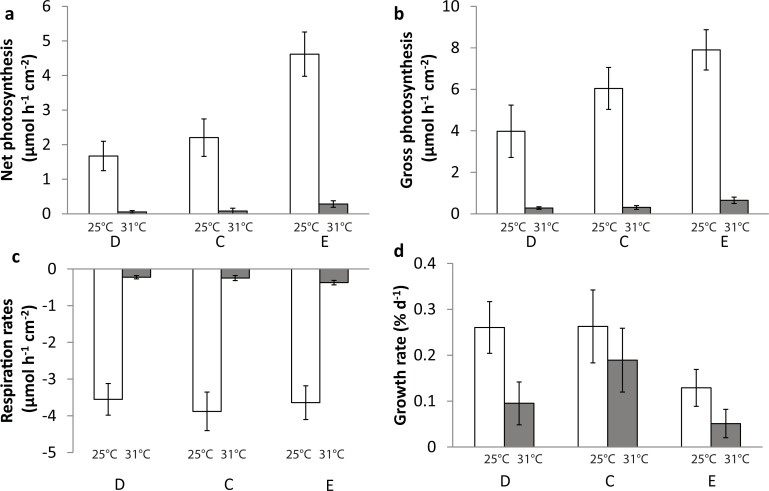
Effect of temperature and nutrient level in absence of UVR exposure. O2 fluxes (μmol O_2_ cm^-2^ h^-1^,) and calcification rates (% d^-1^) in nutrient-depleted (D), control (C) and nutrient enriched (E) condition at 25°C and 31°C. Red numbers in Fig a, b are Pg/R ratio values. Error bars represent standard deviations calculated from five replicates.

For control corals, elevated temperature significantly reduced all symbiont-related parameters (symbiont density, rates of respiration, gross and net photosynthesis) as well as the calcification rates (Figs 2 and 3). In both depleted and nutrient-enriched conditions, elevated temperature was the main parameter explaining the significant decrease in symbiont density (Fig 2A), areal chlorophyll a content (Fig 2B), gross and net photosynthesis rates (Pg and Pn), and respiration rates (R) (Fig 3A, 3B and 3C). Calcification rates were also lower under thermal stress in the nutrient-enriched condition (Fig 3D). Total nitrogen release was slightly but significantly lower under thermal stress in the nutrient-depleted condition. Nutrient level had also significant effect on the coral physiology. Under the normal growth temperature (25°C), nutrient-depletion reduced areal symbiont density and net photosynthesis compared to the control condition (Figs 2 and 3). On the contrary, nutrient enrichment significantly increased the protein content per skeletal surface area (Fig 2B) as well as the net photosynthesis (Fig 3). Overall, Pg:R ratios were higher in the enriched nutrient condition than in control or depleted conditions (Fig 3). Under thermal stress, nutrient depleted corals experienced the highest decrease in chl *a* (per surface area and symbiont cell) and calcification rates. On the contrary, nutrient enrichment had a synergistic positive effect on chl *a*, protein content, Pg, Pn and R, which limited the negative impact of temperature (Figs 2 and 3).

Individual and combined effects of UVR: results of the Generalized Linear Model approach (Tables 2–4) identified an effect of UVR exposure on the symbiont density. However, according to S1 Fig, this effect is unclear, except in the control condition where UVR increased the symbiont density both at 25°C and 31°C (S1 Fig). The lowest rates of gross photosynthesis were obtained in the control condition, at 31°C, under UVR (Pg decreased from 0.36 ± 0.03 μmoles C h^-1^ cm^-2^ without UVR to 0.21± 0.02 μmoles C h^-1^ cm^-2^ under UVR).

### Organic matter fluxes

**Fig 4:**
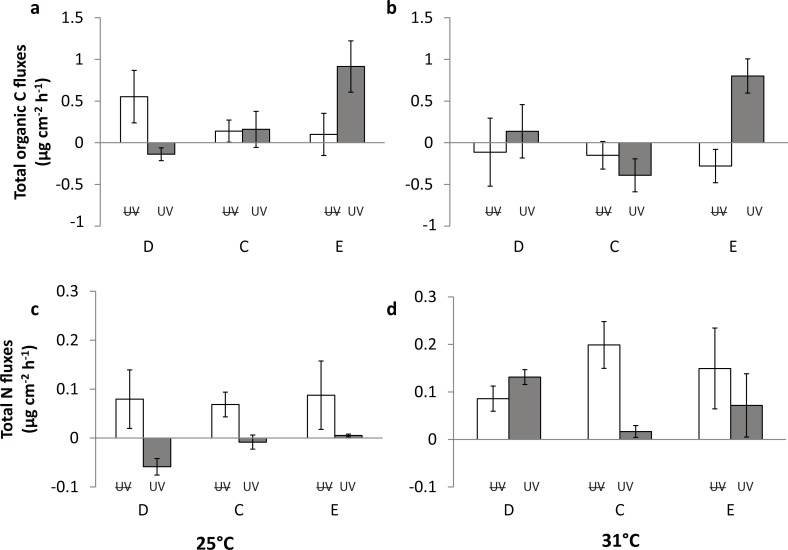
**Total organic carbon (TOC) fluxes (**μ**g cm**^**-2**^ **h**^**-1**^ **) and Total nitrogen (TN) fluxes (**μ**g cm**^**-2**^ **h**^**-1**^ **) in nutrient depleted (D), control (C) and nutrient enriched (E) condition at 25°C (a,c) and 31°C (b,d).** White columns are nubbins growing without UVR; dark grey columns are nubbins growing under UVR. Positive values represent release and negative values represent uptake. Error bars represent standard deviations calculated from five replicates.

Overall, TOC fluxes did not follow any clear trend, due to the numerous interactions between the tested factors (Fig 4A and 4B). The only clear difference is represented by the nutrient-enriched condition, at 31°C with UVR, with a significant release of TOC compared to the other conditions. TN fluxes were more consistent, with a significant decrease in release rates at 25°C under UVR for all nutrient conditions (Table 2, Fig 4C). However, this negative UVR effect was counteracted by elevated temperature in the nutrient enriched and depleted conditions (Fig 4D).

## Discussion

We have examined here the response of the common coral species *Pocillopora damicornis* to the combined effects of three stressors (seawater warming, nutrient enrichment/depletion and UVR exposure), to improve coral reef management. Overall, this study established seawater warming as one of the major stressors impacting the key performance variables of *P*. *damicornis*. The strength of this effect however, is determined by a range of local factors. We have indeed identified increased negative effects of high temperature on certain features of the coral holobiont when combined to nutrient starvation or UVR exposure. Altogether, our results demonstrate that seawater warming will have different effects on shallow and deep corals, which are exposed to different UVR and trophic conditions.

Overall, heat stress led to a significant impairment of the gross and net productivity of *P*. *damicornis*, with a similar decrease in all UVR and nutrient level conditions. In other words, nutrient levels, or UVR exposure, did not increase the severity of bleaching above back ground levels, because temperature had a large impact alone. Two other studies, which have analysed the bleaching severity at different distances from nutrient sources, found no significant impact of nutrient levels on bleaching, in agreement with our observations [3, 52]. It was however observed that high nutrient levels did, to some degree, prolonged bleaching in some coral species, and had a distinct effect on coral microbiomes that was independent of temperature [52]. Many other studies have linked seawater eutrophication with a higher bleaching susceptibility of different coral species [11, 53, 54, 55]. Similarly, coral response to UVR exposure was shown to be species specific and dependent on the duration and/or intensity of the stress (summarized in [56]). All together, these observations suggest that the link between local stressors (such as UVR and nutrients) and temperature is more complicated than originally thought, and may depend, among others, on the severity of the thermal stress. Severe and prolonged warming will mask the effects of local stressors. However, at more moderate levels of heat stress, local environmental conditions, such as nutrient level and UVR exposure can modulate the effect of heat on coral physiology.

Nutrient levels vary in nature with the water quality, the upwellings, the stratification of the water column, the seasonality or the presence of fish and bird colonies on the surrounding islands [57, 58, 59]. Reefs are generally oligotrophic, with ca. 0.5 μM total nitrogen (N) and 0.2 μM reactive phosphorus (P) [60, 61], which is equivalent to our control conditions. During the stratification of the water column, levels of nutrients can decrease to less than 0.2 μM N and un-measurable concentrations of P. However, in many eutrophicated areas, total N can increase to ca. 2–4 μM and even ca. 20 μM, while P vary between 0.3 to 1.5 μM (summarized in [61]). These large changes in nutrient levels may modulate the effect of thermal stress on the main physiological traits of corals. Our results assign a crucial importance to nutrient starvation for coral health. Colonies of *P*. *damicornis* reared in the very low nutrient condition showed a higher bleaching susceptibility, with significant lower symbiont density, chlorophyll content, and rates of photosynthesis compared to nutrient-enriched corals. This impact of nutrient starvation has been observed in few previous studies [62, 63], and support the importance of maintaining a minimum level of nutrients, especially nitrogen and phosphorus, in seawater. Overall, reefs experiencing overfishing, or nutrient shortage due to important water stratification should be particularly susceptible to bleaching during warming episodes.

On the contrary to nutrient-starved colonies, those supplemented with NO_3_^-^ and PO_4_^+^ maintained slightly higher chl *a* and protein content as well as higher photosynthetic rates during thermal stress compared to control colonies. Although it has been shown that nutrient recycling from fish catabolism, or seawater nutrient enrichment can alleviate the negative effects of environmental stress on coral’s photosynthetic capacity [14, 64, 65, 66], many studies have also shown detrimental effects of nutrient enrichment on coral health [11,53, 54, 55]. All together, these observations suggest that the beneficial effect of nutrient supplementation depends on the concentrations and exposure time to the nutrients, on the N:P ratios [27, 67] or on intrinsic physiological characteristics of corals, such as symbiont type or density in coral tissue [68]. Analysis of recent studies points to a relationship between nutrient impact and symbiont density, with a detrimental effect for coral species with symbiont density higher than 10^6^ symbionts cm^-2^ [25, 69], and a beneficial effect for corals with lower symbiont densities, such as in this study [14, 64]. However, correlations between nutrient and temperature need to be further investigated to fully understand their combined effects on coral health.

Apart from nutrient starvation, exposure to UVR worsened the thermal-induced damages on photosynthesis, but only for *P*. *damicornis* colonies maintained under control conditions. This is in agreement with several studies finding synergistic effect of temperature and UVR increases on coral bleaching and mortality [15, 22, 36, 70, 71] and with the general observation that UVR exposure is generally linked to a reduction of the temperature threshold of coral bleaching [15, 23, 72]. However some studies measured no effect, or even a mitigating effect of UV on thermal-stress induced bleaching [22, 73], and the same was observed in this study with corals incubated in depleted and enriched nutrient conditions. All together, these observations suggest that drawing broad conclusions about the combined effects of UVR and temperature on coral bleaching still poses a considerable challenge. Several factors can interact, such as the pre-exposure or acclimation to the stressors [74, 75]; the species genotype [76], or even the nutrient level in seawater (this study) and can mitigate or enhance the bleaching susceptibility of corals exposed to the combined UVR-temperature stress.

Concerning the organic matter fluxes, there was no effect of nutrients and/or temperature on the carbon and nitrogen release rates. A literature review demonstrates that there is no clear relationship between temperature and OM release rates in corals. Some studies observed increased OM release in heat stressed or bleached corals [33, 77,78], while others measured no release [77], or even an uptake of dissolved organic matter [79]. However, UVR significantly changed the quality of the organic matter (OM) released by *P*. *damicornis*, since OM was significantly depleted in nitrogen under UVR exposure. Several factors could have resulted in this observation, such as the impairment in the coral capacity to take up nitrogen, or a different utilization/translocation of nitrogen within the symbiosis, leading to a higher retention in host tissue or symbionts. Although this has never been investigated in corals, and will need more in depth experiments with different coral species, several studies on phytoplankton observed a positive correlation between total nitrogen release and nitrogen uptake [80, 81, 82], as well as a reduction in nitrogen uptake under UVR [67]. Such reduction was attributed to membrane damage and to the inhibition of the enzymes involved in nitrogen metabolism [83, 84, 85]. If this UVR effect is repeatedly observed in corals, elevation in UVR levels on reefs is thus expected to generate products with high C:N molar ratio, i.e. enriched in carbon and depleted in nitrogen. During OM regeneration, bacteria will likely rapidly take up the small amounts of nitrogen available, leading to a depletion (or possibly starvation) of bioavailable nitrogen in surface reef waters. These preliminary observations suggest that future UVR and temperature-induced changes in the quality of the organic matter released by corals, combined with other changes in microbial nutrient recycling, will induce dramatic changes in the seawater C:N:P ratios in surface reef waters. These changes can lead to imbalance nutrient ratios that will have considerable impact on the ecology of coral reefs from the micro to the macro scale. However, more data are needed to fully understand the impact of environmental changes on the biogeochemical cycles. For instance, N_2_ fixation by corals and other benthic functional groups, such as algal turfs [86] may increase in the future under heat exposure, following an increase in diazotroph community size and activity [87]. Elevated N_2_ fixation rates are already observed in summer compared to winter [88, 89]. This increase N_2_ fixation may counterbalance the decreased nitrogen availability through coral OM production and bacterial remineralization.

One of the most significant challenges now for the scientific community is to identify the factors that promote or worsen coral’s resilience to global warming. This is because interactions among several stressors, where the effect of one is dependent on the magnitude of another, are very common in nature [90]. The results obtained in this study, confirm those obtained in a recent meta-analysis [3], and show that seawater warming is the main factor impacting coral health. Therefore, during severe heat-stress, other factors such as UVR exposure, nutrient availability and water quality affords little resistance to bleaching. These factors can however become important during mild episodes of heat stress, or for the recovery of corals after warming episodes. Our study also highlights a significant effect of UVR and high temperature on the quality and degradation of the organic matter released by corals into reef waters. Overall, both UVR and seawater warming will induce changes in the seawater C:N:P ratios in surface reef waters, which in turn will have considerable impact on the ecology of coral reefs.

## Supporting information

S1 Fig **Effect of UVR on a) the symbiont density at 25°C; b) the symbiont density at 31°C; 3) the gross photosynthesis at 31°C**. White and black bars represent the no UVR and UVR condition, respectively.
(EPS)
